# Prostate Artery Embolization—Review of Indications, Patient Selection, Techniques and Results

**DOI:** 10.3390/jcm10215139

**Published:** 2021-10-31

**Authors:** Sailendra G. Naidu, Harish Narayanan, Gia Saini, Nicole Segaran, Sadeer J. Alzubaidi, Indravadan J. Patel, Rahmi Oklu

**Affiliations:** 1 Division of Vascular & Interventional Radiology, Mayo Clinic Arizona, Phoenix, AZ 85054, USA; narayanan.harish@mayo.edu (H.N.); alzubaidi.sadeer@mayo.edu (S.J.A.); patel.indravadan@mayo.edu (I.J.P.); oklu.rahmi@mayo.edu (R.O.); 2 Division of Vascular & Interventional Radiology, Laboratory for Patient Inspired Engineering, Mayo Clinic, Phoenix, AZ 85054, USA; saini.gia@mayo.edu (G.S.); nsegaran2@gmail.com (N.S.)

**Keywords:** prostate artery embolization, benign prostatic hypertrophy, lower urinary tract symptoms, hematuria, nocturia

## Abstract

Lower urinary tract symptoms (LUTS) due to benign prostatic hypertrophy (BPH) are a very common problem in men ranging from mild urinary symptoms to recurrent urinary tract infections or renal failure. Numerous treatment options are available ranging from conservative medical therapies to more invasive surgical options. Prostate artery embolization (PAE) has emerged as a novel treatment option for this common problem with clinical efficacy comparable to the current surgical gold standard, transurethral resection of the prostate (TURP). PAE offers fewer complications and side effects without a need for general anesthesia or hospitalization. This review discusses the indications for prostate artery embolization in addition to LUTS, patient evaluation in patients with LUTS, PAE technique and clinical results, with an emphasis on efficacy and safety.

## 1. Introduction

Lower urinary tract symptoms (LUTS) due to benign prostatic hypertrophy (BPH) is a common problem affecting more than 20% of men between the ages of 30 and 79. The prevalence increases with age; approximately 80% of men are affected by BPH symptoms by 70 years of age [1]. Conservative therapies such as lifestyle modifications or medical management are generally first line therapies. In patients who do not respond sufficiently, or have any evidence of renal insufficiency, urinary retention, recurrent urinary tract infections or hematuria, other forms of treatment are considered. Numerous treatment options exist including transurethral resection of the prostate (TURP), transurethral vaporization of the prostate (TUVP), prostatic urethral lift (PUL), amongst others [2]. Table 1 introduces some of the common surgical approaches used to treat BPH, including descriptions of each therapy as well as which therapies are indicated for different prostate sizes. TURP is considered the surgical gold standard for treating LUTS related to BPH.

Recently, prostate artery embolization (PAE) has emerged as an alternative treatment option for LUTS, falling between medical management and surgical options in the spectrum of BPH therapy. In 2018, the United Kingdom National Institute for Health and Care Excellence (NICE) found the safety profile and efficacy to be adequate to support PAE in properly selected patients [11]. The advantages of PAE include no hospitalization, no general anesthesia, avoidance of the potential risks associated with surgery and a decreased risk of sexual health side effects such as retrograde ejaculation or erectile dysfunction [12,13].

This article will go over the indications for PAE as well as the pre-procedural evaluation process. Patient selection criteria is critical and the key factors in the evaluation including clinical workup will be reviewed. This is followed by a detailed techniques section of the prostate artery embolization procedure as performed at the author’s institution. The clinical efficacy, safety profile and potential complications of PAE will be evaluated using a narrative review of various retrospective cases, randomized controlled trials, and meta-analyses studies that have been published within the past decade. Lastly, the future directions and potential refinements in the PAE procedure will be discussed.

## 2. Indications

Although no clear guidelines are identified for PAE, the most common indication is symptomatic men who are considering treatment of LUTS [14,15]. A more in-depth review on this indication for PAE will be covered in the pre-procedural evaluation section.

PAE can be used in treatment of refractory hematuria of prostatic origin (RHPO) due to prostate cancer or BPH [16]. Complete urologic examination and consultation is necessary to exclude other potential causes of hematuria. Clinically, prostatic hematuria due to prostate cancer is challenging to treat and often managed initially with continuous bladder irrigation. In more advanced cases, cystoscopic transurethral treatments may be necessary. One study evaluated refractory hematuria in 9 prostate cancer patients who had previously undergone bladder irrigation and cystoscopic intervention. Six out of nine (67%) of patients had resolution of their hematuria following PAE [17]. In another study, twenty patients with hematuria due to BPH underwent PAE. This demonstrated resolution of the hematuria in 85% of patients at 3 months and 80% at 12 months [18]. In a different study evaluating gross hematuria in BPH patients, 34 out of 37 (92%) patients were hematuria-free 483 ± 137 days after undergoing PAE [19].

PAE can also be a palliative procedure for prostate cancer patients with LUTS or catheter dependent urinary retention. In a small study of advanced prostate cancer patients with significant LUTS who were not surgical candidates, PAE was effective at decreasing their International Prostate Symptom Score (IPSS) by a mean of 12.2 points in 5 patients [20].

Patients with chronic indwelling catheters due to BPH may also benefit from PAE. Many of these patients are not good surgical candidates and surgical treatment options may be limited. Long standing indwelling catheter use significantly affects a patient’s quality of life and activities of daily living. Multiple studies have shown PAE to be a safe and effective treatment for catheter dependent BPH with significant improvement in quality of life [21]. One study evaluated 43 catheter dependent patients who were not surgical candidates following PAE. Catheter removal was achieved in 33 (81%) of patients [22]. In another study, similar results were found, with 80% of catheter-dependent becoming catheter-free 3 months after undergoing PAE [23]. Comparable results were found in another study showing 33 out of 38 (87%) of catheter-dependent patients capable of being catheter-free after 3 months [19].

Iatrogenic hemorrhage after TURP or urologic intervention is a rare complication. First line treatment for iatrogenic hemorrhage after surgery is conservative management. In patients who have failed initial conservative medical management, PAE has been demonstrated to be a reasonable and effective alternative. This may be true especially when excessive bleeding prevents adequate visualization of the source under cystoscopy [24].

Finally, for patients who have failed transurethral interventions for BPH, prostate artery embolization is a viable treatment option. One study demonstrated positive outcomes 3 months after prostate artery embolization on three patients who failed transurethral intervention initially. These patients displayed a reduction of IPSS score by a mean of 13.7, as well as a reduction of prostate gland size by about 32% [25]. PAE can still be offered after urologic therapies.

## 3. Pre-Procedural Patient Evaluation

All potential PAE patients are seen in clinic prior to scheduling the prostate artery embolization procedure. Identifying patients who will benefit from PAE is critical as there are factors that could decrease chance of clinical success. It is often recommended that patients be seen by both urology and interventional radiology to go over all potential treatment options and to exclude other causes of LUTS [26,27,28]. Identifying patients with LUTS due to BPH is important as there are numerous potential confounding causes of these symptoms. For example, patients may have detrusor dysfunction, neurogenic bladder, overactive bladder, or a urethral web/structure. These patients may not gain a benefit from PAE as the underlying problem is not due to prostatic obstruction. 

The International Prostate Symptom Score (IPSS) is a validated questionnaire used to assess the severity of symptoms. This score is useful to both identify patients who may benefit from a given intervention and assess response to therapy. This symptom score is the most widely utilized in the BPH literature. The questionnaire is made of up 7 questions each with a score of 0–5 (with a maximum score of 35) depending on the symptom severity (i.e., incomplete emptying, frequency, intermittency, urgency, weak stream, straining and nocturia). A total score of 0–7 indicates mild disease and a score of 8–19 represents moderate disease, while a score above 20 indicates severe disease. The last question on the IPSS is a quality-of-life question, which is an overall self-assessment of symptoms ranging from 0 (delighted) to 6 (terrible) [29,30]. While there is no consensus, in general, the baseline inclusion criteria in multiple studies is a symptom score above 13 to justify treatment [31,32,33]. It may also be useful for patients to complete the International Index of Erectile Function (IIEF) to determine the degree, if any, of erectile dysfunction [28,29].

Many treatment options for LUTS due to BPH may have sexual side effects [34]. This can significantly affect decision making when choosing which form of therapy to pursue. Overall, PAE is not associated with worsening erectile function; in fact, some patients may show improvement. In a retrospective study with 630 patients, the IIEF improved an average of 1.2 ± 5.74 points (*p* = 0.0003) with 64% of patients showing improvement or no change in the IIEF score [12]. In terms of ejaculatory dysfunction, TURP has a reported incidence of retrograde ejaculation of around 70% [35]. However, newer techniques in TURP have been developed that can preserve the sphincter and may lower this incidence. The UK-ROPE study found rates of retrograde ejaculation of 24.1% following PAE and 47.5% in the TURP group [36]. This study was limited by lack of baseline patient data, many of whom were on medication, which may have induced retrograde ejaculation prior to the interventions. One retrospective PAE single center study with 152 patients had no reported cases of retrograde ejaculation [37]. The overall risk of retrograde ejaculation following PAE based on meta-analyses is estimated to be 0–2.3% [38]. Other sexual side effects from PAE such as hematospermia can occur in 16% of patients but they are generally transient [34].

IPSS symptoms are divided into voiding symptoms (i.e., incomplete emptying, intermittency, weak stream, straining) and storage symptoms (frequency, urgency and nocturia). When voiding symptoms are responsible for a greater proportion of the elevated IPSS score, the findings are more likely due to BPH. Conversely, if storage symptoms are elevating IPSS score, other potential causes of LUTS should be considered [39]. Such patients may benefit from further urologic evaluation such as urodynamic testing. Differentiating between the two subcategories can also have some prognostic value in determining who will benefit from PAE. One study did show a benefit in all IPSS symptoms; however, greater benefits were seen in patients with predominant voiding symptoms [40].

Useful laboratory values include a complete blood count (CBC), creatinine, and prostate-specific antigen (PSA) [41]. Elevated PSA levels should prompt further evaluation to exclude the possibility of prostate cancer. A prostate biopsy can be performed as clinically warranted [28,42].

Following the collection of laboratory data, a review of medications should be conducted [28]. Many patients have undergone a trial with medications to help with LUTS. These should be held for at least one week prior to the scheduled PAE. The use of erectile dysfunction medications should be noted and withheld prior to the procedure for at least three days, should intra-arterial nitroglycerin be used during the PAE procedure.

While most patients often have had a transrectal ultrasound (US) of the prostate when presenting for consultation, additional cross-sectional imaging should be obtained using either contrast-enhanced CT or MR. Both imaging modalities are useful for assessing the degree of tortuosity, calcifications and any stenosis within the iliac arteries that can make the PAE more complicated. Finally, the prostate volume (PV) can be calculated from cross-sectional imaging.

**Figure 1:**
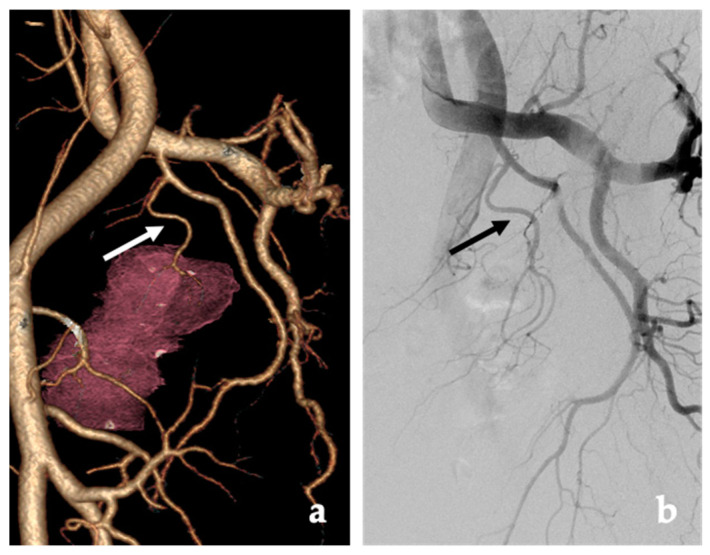
Computed Tomography Angiography (CTA) is useful for PAE procedural planning. (**a**) CTA 3-D reconstruction using the dedicated prostate artery protocol demonstrates the prostate artery origin arising from the internal pudendal artery (white arrow). (**b**) Digital subtraction angiography (DSA) imaging performed at 35 degrees LAO projection shows the prostatic artery arising from the internal pudendal artery (black arrow) correlating with the CTA.

Many prefer the use of CT due to the speed of acquisition, spatial resolution and the ability to visualize very small arteries (Figure 1) [14,27,43]. CT angiography may also help identify the origins of the prostatic artery, which can be one of the more challenging steps in the procedure [27].

CT scan protocols can vary based on equipment as well as by institutional protocols. The authors’ workflow and scanning parameters are as follows. 800 mcg nitroglycerin is administered via a spray form under the tongue five minutes prior to scanning [28]. Intravenous (IV) injection of intravenous iohexol (Omnipaque 350, GE Healthcare, Waukesha, WI, USA) is administered at a rate of 5 mL/s scanning from a level 10 cm above the iliac crest to the lesser trochanter. The amount of contrast administered is based on weight with 1 mL contrast injected per every pound of body weight (up to maximum 200 mL). Following contrast administration, 30 mL of saline is used as a flush [44]. Contrast bolus tracking software is used and triggers scanning when the Hounsfield (HU) at the level of the infrarenal aorta (L3 vertebral body) reaches 300 HU. Scan parameters are as follows: 0.6-mm collimation, pitch 0.6, tube voltage 120 kV, 250 reference mA with automated tube modulation based on body habitus (CARE Dose 4D, Siemens Medical Systems). Venous phase imaging is also obtained 70 s after the arterial phase is performed. Coronal, sagittal and 3D volume rendering technique (VRT) reconstructions are made. 3D reconstructions are made using either GE (GE Healthcare, Waukesha, WI, USA) or TeraRecon (TeraRecon, Durham, NC, USA) imaging software.

Prostate size can be an important consideration in deciding who may benefit from PAE; there have been mixed results in the literature on this topic. A multivariate study looking at clinical response to PAE as measured with the IPSS found prostate size to have a significant association with clinical improvement [45]. Another study compared large prostate size (>80 g) and medium prostate size (50–80 g) to determine if pre-procedure prostate size has an impact on clinical outcomes [46]. They found both groups to have significant improvements in the IPSS, post-void residual (PVR) and PV. However, the large prostate size group had significantly better outcomes compared to the medium prostate group with regards to IPSS, maximum flow rate (Q_max_), PV and PVR. Several authors consider PAE to be better suited for larger prostates (>80 g) as it may be more technically amenable to embolization and more likely to have favorable results compared to smaller prostates (<50 g) [28,47,48]. Other studies have shown little correlation with prostate size and clinical outcome; one retrospective study evaluated prostate size prior to PAE in order to determine its impact on IPSS score. Groups were divided into 3 prostate sizes: <50 g, 50–80 g and >80 g. All groups demonstrated a significant improvement in IPSS with no difference between the three groups [3]. When prostate size is below 40 g, the patient should be counseled regarding this finding in the decision-making process. This limitation is an important factor to be considered particularly when contemplating other available potential treatment options. 

Prostate lobe morphology may have prognostic implications. Intravesical prostatic protrusion (IPP), often used synonymously with median lobe hypertrophy may cause a ‘ball valve’ type of obstruction, which could potential worsen with PAE due to the softening of the prostate. A prospective study compared the outcomes following PAE in patients with IPP compared to those without [49]. The presence of IPP was significantly associated with negative outcomes with higher rates of acute urinary retention or worsening LUTS. However, when the IPP was divided into two groups; those with a thickness-to-height (T/H) ratio <1.3 and those with a T/H ratio >1.3, significant differences were present. Those with a T/H ratio <1.3 (more ‘tubular’ prostatic impression on the bladder) had significantly more complications and worse clinical outcomes. Therefore, the degree and morphology of IPP may be more important than simply for determining whether IPP is present [50].

## 4. Techniques

### 4.1. Preprocedure

Prior to the procedure, a prophylactic dose of antibiotics is given (often either ciprofloxacin (400 mg) or ceftriaxone (2 g) via IV) [28,48,51]. Fluids (0.9% NS) are run at 100 mL/h throughout the procedure. Ketorolac (30 mg) or another nonsteroidal anti-inflammatory (NSAID) can be administered one time as an anti-inflammatory to decrease post-embolization discomfort [28]. The procedure is performed with moderate conscious sedation. If a Foley catheter is going to be placed, this is performed in the IR suite after sedation has begun. A Foley catheter can be useful for prostate localization and may be beneficial for long procedure times, however, it is not necessary [28]. The Foley balloon is filled with dilute contrast solution (1:10) such that it can be identified with fluoroscopy [52].

### 4.2. Procedure

Prostate artery embolization can be performed from a femoral or radial approach [28]. If the femoral artery is accessed, the contralateral internal iliac artery is selected using a 5-French catheter. Cobra shaped catheters are often preferred, but an angled catheter can be utilized as well [53]. In patients with extensive iliac tortuosity, a sheath may be placed over the iliac bifurcation into the contralateral common iliac artery. This will provide support and facilitate cannulation of the internal iliac artery. Digital subtraction angiography (DSA) imaging is performed to visualize the blood vessels and blood flow. To visualize the left internal iliac artery, DSA is conducted via the left anterior oblique (LAO) with a 35-degree ipsilateral projection and 5–10 degrees of caudal angulation. If pre-procedural imaging is available, this is reviewed prior to the procedure to reduce the number of digital subtraction acquisitions required. If the prostate artery is not well identified or the origin is difficult to find on the initial DSA run, an additional angiogram can be performed at 45 degrees. If the origin of the prostate artery is still not clear, a cone beam CT can be performed to help identify the prostatic artery [26,27,43]. An injection of 50% diluted contrast is power injected via the 5F catheter within the internal iliac artery at a rate of 4 mL/sec for a total of 16 mL. Another option is to begin the case with a cone beam CT of the pelvis using a pigtail catheter in the aorta. This allows for bilateral visualization of the iliac artery anatomy with one cone beam acquisition reducing the number of required runs [54].

**Figure 2:**
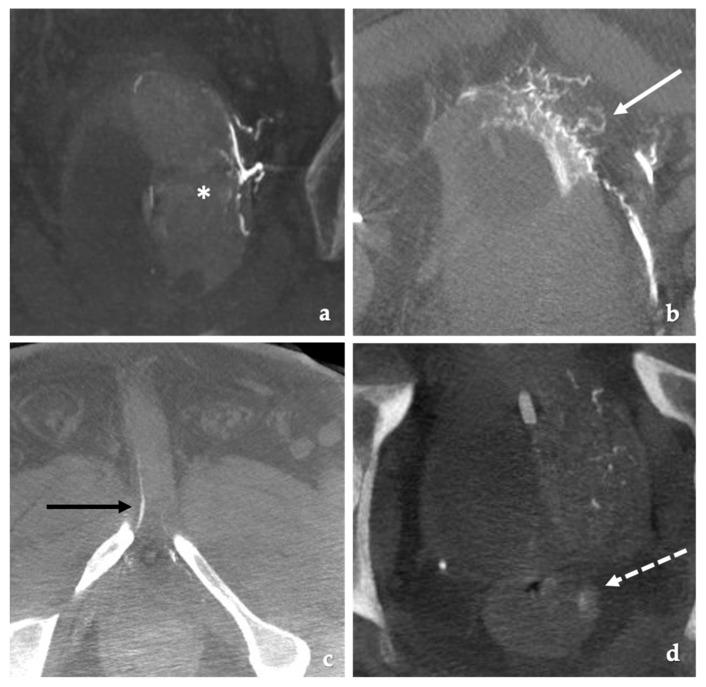
Examples of cone beam CT demonstrating target and non-target embolization. (**a**) Coronal reconstruction from a cone beam CT acquisition. Appropriate contrast enhancement of the left hemiprostate (white asterisks) with contrast injection of the left prostatic artery. (**b**) Axial cone beam CT image. Non-target filling of the bladder (white arrow). Foley catheter balloon in the bladder. (**c**) Axial cone beam CT image. Non-target filling of the penile artery (black arrow). (**d**) Axial cone beam image. Non-target enhancement of the rectum (dotted white arrow) with contrast enhancement of the prostate.

After identification, a 0.020” microcatheter is advanced into the prostate artery using a 0.014” wire. DSA imaging is then performed in the AP projection to identify and confirm filling of the prostate and evaluate for any sites of non-target embolization (Figure 2) [55]. Potential sites include the bladder, seminal vesicles, rectum and the penis. Cone beam CT or rotation angiography may be used to confirm no evidence of non-target embolization and to ensure the entire hemiprostate shows enhancement [26,27,43]. When using cone beam CT, the injection needs to be done very slowly to minimize reflux and contrast filling of more proximal branches thereby giving false positive sites of non-target embolization. Power injection at rates as low as 0.3 mL/s is performed [28]. Hand injection with the operator behind a floor mounted lead shield is another option. 

After confirming appropriate placement, embolization is performed using calibrated tris-acryl gelatin microspheres (Embospheres; Merit Medical, South Jordan, UT, USA) in the 300–500 μm size range. While 100–300 μm beads can be used, one comparative study showed no significant difference in clinical efficacy between the two sizes, however, the 100–300 μm particle group displayed increased adverse events [56]. The particles are mixed in a dilute saline and contrast solution such that the beads are well suspended. The beads should be infused very slowly in one cc aliquots with intermittent 1–2 mL saline flushes between each administration [28]. Infusion should be done under real time fluoroscopy to decrease chance of reflux.

The PErFecTED (Proximal Embolization First Than Embolize Distal) technique is a further refinement on PAE technique that can be used to potentially increase the degree of infarction and ischemia [52]. After embolization is performed from a proximal site as described above, the microcatheter is further advanced into the prostate and additional particulate embolization performed. Studies have shown increased infarction and improved clinical results over conventional PAE when using this technique [55].

**Figure 3:**
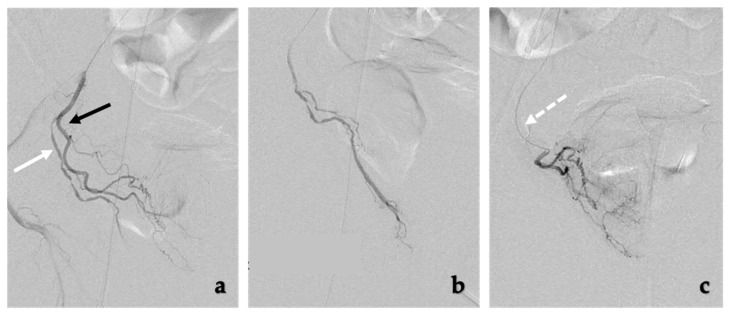
Coil embolization of extra-prostatic arteries. (**a**) The middle rectal artery (white arrow) and prostatic artery (black arrow) share a common trunk with short distance between middle rectal artery and prostatic artery. To safely embolize the prostatic artery with particles, the middle rectal artery can be embolized with coils, (**b**) Selective injection of the middle rectal artery prior to coil embolization, (**c**) DSA imaging of the prostatic artery after coil embolization (white dotted arrow) of the middle rectal artery shows filling of the prostate without filling of the rectum. Following these steps, the prostatic artery can now safely be embolized with particles.

The prostate arteries are very small and prone to vasospasm upon manipulation [43]. Nitroglycerin should be readily available on the Table 2 and can be given intra-arterially as needed. Additionally, coil embolization may need to be performed of any non-target arteries, generally to the rectum or penis (Figure 3). Coil embolization may be needed in about 26% of all cases and can prevent particles from distal embolization [57].

After embolization of the contralateral side, a Waltman loop is formed with the C2 catheter and the ipsilateral internal iliac artery is selected [53]. Alternatively, a reverse curve catheter may be utilized to cannulate the ipsilateral internal iliac artery [53,54]. DSA imaging is performed from the internal iliac artery and the same steps are performed. 

### 4.3. Post-Procedure

Immediately following the procedure, if a Foley catheter was placed, this is removed. The patient must be able to void prior to discharge. Urinary retention following the procedure is likely due to prostate inflammation and is known to occur in approximately 5–8% of patients [26,58,59]. If the patient is unable to void, a Foley catheter is replaced and removed later, often allowing up to one or two weeks for the inflammation to subside [28].

The procedure is performed on an outpatient basis. Prescriptions are given for antibiotics and NSAIDs following the procedure [28]. Additionally, urinary analgesics can be taken with any symptoms of dysuria, and laxatives are recommended if there is any discomfort with defecation [60].

## 5. Outcomes

Metrics used to evaluate the clinical efficacy of procedures for the treatment of LUTS include subjective self-assessment tools including IPSS, IIEF and quality of life (QOL) scores described above. Objective outcomes used in most studies include PV, PVR and Q_max_, assessed before and after a given intervention. These studies can be divided into retrospective or prospective case series, randomized controlled trials comparing PAE to TURP (the surgical gold standard) and meta-analyses.

### 5.1. Prospective and Retrospective Case Series

There have been numerous retrospective or prospective case series from multiple international sites demonstrating the efficacy of PAE for the treatment of LUTS [3,40,61,62,63,64,65]. There were slight variations in methodology and follow up per study, but each demonstrated good clinical outcomes overall. 

One of the largest retrospective studies included 630 patients with short (<1 year), medium (1–3 years) and long term (3–6.5 years) clinical follow up for PAE patients [12]. The mean change from IPSS baseline was −13.7–14.5 and −16.9 in short, medium and long term, respectively. The Kaplan-Meier estimates of cumulative rates of clinic success were 85.1%, 81.9% and 76.3%, respectively. This study demonstrated the lasting effect of PAE with good long term follow up.

Another large retrospective single center study, which included 317 patients over a 10 year experience, had long term data with a median follow up of 72 months [53]. The mean IPSS reduction score was 16 ± 7 points and the mean QOL score reduction score was 4 points. Symptom recurrence occurred in 23% of patients.

A multi-center registry, the UK Register of Prostate Embolization (UK-ROPE), recruited 305 patients of which 216 underwent PAE. A propensity-matched comparison was made with 89 patients who underwent TURP [36]. The study showed a median 10-point reduction in the IPSS score 12 months after PAE compared to the baseline. While statistically significant, this was less effective compared to TURP, which produced a median 15-point reduction at the same time interval. Objective evaluation demonstrated an improvement in Q_max_ by 3 mL/s and a decrease in PV by 25 mL (27.8%) at a 12-month interval. This study utilized the Clavien-Dindo classification system to determine the severity of surgical complications. Grade I-II represent minor, common complications that can be resolved with pharmacological treatment while Grade III-IV represent major, severe complications that may require surgery or be life-threatening [66]. No major complications of Clavien Grade ≥ 3 were reported in neither the PAE group nor the TURP group. The overall reoperation rates were higher in the PAE group (19.9%) compared to the TURP group (5.6%).

### 5.2. Randomized Controlled Trials

There have been a few prospective randomized trials comparing PAE to TURP. The first was a study with 114 patients whereby 57 patients were randomized to each group [67]. Patients were followed for 2 years and assessments of IPSS, QOL, Q_max_ and PVR were recorded. Both therapies showed significant reduction in the IPSS score from baseline; however, the TURP group had a more rapid improvement at a one-month interval (−11.0 reduction in TURP vs. −5.1 reduction in PAE). At the one-year interval mark, the reductions were more similar (−14.5 reduction in TURP vs. −13.4 reduction in PAE). Other parameters showed similar findings with comparable results noted at the 1- and 2-year time points. Additionally, this study also utilized the Clavien-Dindo classification to categorize the surgical complications. The study did show higher major adverse complications in the PAE group compared to the TURP group; however, both post-embolization syndrome and acute urinary retention were included as minor complications. Technical and clinical treatment failures, which were more common in the PAE group, were included as major adverse events.

Another prospective randomized trial compared original PAE (oPAE) to TURP with 15 patients in each group [55]. Both groups were then compared to a third group utilizing the PErFecTED modification PAE. All groups had significant improvement in IPSS, QOL and Q_max_. The degree of improvement was highest in the TURP, and PErFecTED techniques compared to oPAE (−21.5 in TURP, −21.0 with PErFecTED technique and −12.5 in oPAE). The adverse events were higher in the TURP group compared to both PAE groups.

A prospective study was performed randomizing 48 patients to PAE and 51 patients to TURP with primary endpoints evaluated at 12 weeks [68]. The mean reduction in IPSS from baseline to 12 weeks was similar between the groups (−9.23 in the PAE group and −10.77 in the TURP group). However, PAE was less effective at improving functioning outcomes such as Q_max_ (5.19 mL/s improvement in PAE vs. 15.34 mL/s improvement in TURP). In addition, this study also utilized the Clavien classification system to determine the severity of surgical complications and reported more Clavien Grade ≥ 3 treatment related adverse effects in the TURP group when compared to the PAE group 

A recent randomized control study prospectively enrolled 23 patients to PAE and 22 patients to TURP [69]. The primary outcomes were assessed by Q_max_ and IPSS at 12 months. The mean IPSS reduction at 12 months was 21.0 in the PAE group and 18.2 in the TURP group. The mean Q_max_ increased from 6.1 mL/s in the PAE group compared to 9.6 mL/s in the TURP group. The TURP group displayed more treatment related complications, with adverse events graded via the Clavien classification system.

To address the question regarding a possible placebo effect from those undergoing PAE, a randomized, single-blind clinical trial was performed in 80 men comparing a sham procedure to PAE. After successful catheterization of a prostate artery, patients were randomized into the sham group (no embolization procedure performed) and conventional PAE group [70]. Coprimary outcomes were the change from baseline of IPSS and QOL score at 6 months. The decrease in IPSS from baseline was 5.03 ± 8.13 in the sham group compared to 17.1 ± 7.25 in the PAE group at the 6-month interval (difference of decrease in IPSS score between the groups: 13.2, 95% CI 10.2–16.2, *p* < 0.0001). The study had an open extension phase at the 6 month point whereby patients in the sham group underwent PAE with another evaluation 6 months later (12 months from baseline). Following PAE in the sham patients, the IPSS scores decreased by 13.6 ± 9.19 (*p* < 0.0001). When compared to the patients who underwent PAE at baseline (12 month follow up), the IPSS scores were comparable in both groups (9.0 in PAE group and 8.6 in the sham + subsequent PAE group), therefore demonstrating the positive treatment effect of PAE compared to a sham procedure.

### 5.3. Meta-Analyses

Several meta-analyses have been performed combining the results of published series to evaluate the efficacy of PAE [59,71]. One of the first was published in 2016 where exclusionary criteria was used for the analysis [58]. To conduct the analysis, 19 studies were selected for the data collection, while only 6 studies included enough data to be included in the meta-analysis. At 12 months, the IPSS showed improvement of 20.39 points from baseline and the QOL score had improved by 2.49 points. 

One meta-analysis published in 2018 found 13 studies meeting the inclusion criteria with a total of 1254 patients presenting with BPH and LUTS [26]. The reduction in the IPSS score was 16.2 points at a 12-month interval and the QOL score had decreased by 3.0 points at the same interval.

**Table 2:** A summary of the retrospective studies, randomized controlled trials and meta-analyses studies. A narrative review was performed to determine the safety and efficacy of PAE. Data is presented as the reported mean differences from the baseline values.

Author	Type of Study	Follow-Up Duration	IPSS (Points) Improvement	QOL (Points) Improvement	Q_max_ (mL/s) Improvement	PV (cm^3^) Reduction	Number of Patients Analyzed	Major Reported Complications
Pisco et al. [12]	Retrospective	3–6.5 years	PAE: −16.94 ± 8.70	PAE: −1.74 ± 1.45	PAE: 7.98 ± 4.83	PAE: −16.85 ± 25.70	PAE: 630	PAE: 2 (bladder ischemia, persistent perineal pain)
Carnevale et al. [53]	Retrospective	3 months–8 years	PAE: −16 ± 7	PAE: −4 ± 1	PAE: 6 ± 10	PAE: −39 ± 39	PAE: 317	PAE: 5 (persistent UTI, collapsed asymmetric median lobe, bladder ischemia)
Ray et al. [36]	Retrospective	1 year	PAE: −10.9 TURP: −15.2	PAE: −2.6 TURP: −3.4	PAE: 4.4 TURP: 8.6	PAE: −28.6 TURP: Not measured	PAE: 216 TURP: 89	PAE: 0 (Clavien Grade ≥ 3 complications) TURP: 0 (Clavien Grade ≥ 3 complications)
Gao et al. [67]	Randomized controlled	2 years	PAE: −15.6 TURP: −16.3	PAE: −3.2 TURP: −3.2	PAE: 13.7 TURP: 14.8	PAE: −29.8 TURP: −36.9	PAE: 57 TURP: 57	PAE: 8 (technical and clinical failures) TURP: 4 (transurethral resection syndrome, clinical failures, bladder neck stenosis)
Carnevale et al. [55]	Randomized controlled	1 year	oPAE: −12.5 PErFecTED: −21.0 TURP: −21.5	oPAE: −2.5 PErFecTED: −3.1 TURP: −3.7	oPAE: 3.1 PErFecTED: 11.6 TURP: 17.4	oPAE: −12.1 PErFecTED: −16.2 TURP: −24.6	oPAE: 15 PErFecTED: 15 TURP: 15	oPAE: 0 PErFecTED: 0 TURP: 2 (left venous sinus intra-operative damage and rupture of prostatic capsule, bladder catheterization and temporary irrigation)
Abt et al. [68]	Randomized controlled	12 weeks	PAE: −9.23 TURP: −10.77	PAE: −2.33 TURP: −2.69	PAE: 5.19 TURP: 15.34	PAE: −12.17 TURP: −30.27	PAE: 48 TURP: 51	PAE: 2 (Clavien Grade ≥ 3 complications) TURP: 7 (Clavien Grade ≥ 3 complications)
Insausti et al. [69]	Randomized controlled	1 year	PAE: −21.0 TURP: −18.2	PAE: −3.78 TURP: −3.09	PAE: 6.1 TURP: 9.6	PAE: −20.5 TURP: −44.7	PAE: 23 TURP: 22	PAE: 0 (Clavien Grade ≥ 3 complications) TURP: 1 (Clavien Grade ≥ 3 complication)
Pisco et al. [70]	Randomized controlled	6 months	PAE: −17.1 ± 7.25	PAE: −3.00 ± 1.13	PAE: 6.82 ± 6.25	PAE: −17.6 ± 18.5	PAE: 39	PAE: 1 (Clavien Grade ≥ 3 complication)
Uflacker et al. [58]	Meta-analysis	1 year	PAE: −20.39	PAE: −2.49	PAE: 5.39	PAE: −31.31	PAE: 662	PAE: 2 (bladder ischemia, transient ischemic rectitis)
Malling et al. [26]	Meta-analysis	1 year	PAE: −16.2	PAE: −3.0	PAE: 6.5	PAE: -20.3	PAE: 1046	PAE: 3 (bladder ischemia, persistent UTI, persistent perineal pain)

## 6. Safety and Complications

Complications from PAE are rare with most representing minor complications. Common minor complications include dysuria, hematospermia, urinary retention and urinary tract infections [72,73]. The 2016 meta-analysis comprised of nine studies and 662 patients demonstrated an overall complication rate of 32.9% with 99% related to “minor complications” or SIR class A/B complications [58]. The most frequent minor complications noted in this meta-analysis were rectalgia/dysuria (9.1%), acute transient urinary retention (7.9%) and transient hematuria (4.4%). Another meta-analysis found that the most common minor complications were dysuria (17.0%) and transient increased urinary frequency (11.6%) [26]. 

Major complications associated with prostate artery embolization are generally due to nontarget ischemia to the bladder, rectum or penis. The 2018 meta-analysis found 3/1253 (0.3%) patients displayed major complications [26]. This included bladder wall ischemia requiring cystoscopic treatment, urinary tract infection requiring IV antibiotics and persistent perineal pain. Other less common major complications related to PAE include prostatic abscess or the detachment and expulsion of prostatic tissue via the urethra with subsequent bladder obstruction [73,74]. Penile ulcers can also occur, which are thought to be secondary to ischemic effects of non-target embolization. Overall, prostate artery embolization is a safe procedure with the vast majority of complications representing self-limited minor complications.

Review of comparative trials between PAE and TURP demonstrate a lower rate of major complications when using PAE. For example, a prospective randomized control trial found that PAE resulted in half the number of complications when compared to TURP after 12 months (36 adverse events vs. 70 adverse events; *p* = 0.003) with the most common being pain and urinary tract infection [68]. In another randomized control trial, fewer adverse events were reported in the PAE group compared to the TURP group (n = 15 vs. n = 47; *p* < 0.001) [69]. A propensity matched study found PAE patients were able to return to normal activities more quickly than TURP patients (mean of 5 days vs. 14 days), while also exhibiting a reduced length of hospital stay or need of admission compared to TURP [36].

## 7. Future Directions

An area of interest in prostate artery embolization is drug delivery to potentiate the effects of embolization. Using the properties of bleomycin as a sclerosing agent, one study evaluated bleomycin-eluting microspheres compared to bland microspheres in a canine model [75]. This comparative animal study showed greater prostate volume reduction in the bleomycin group compared to the bland group (74.1% ± 4.3 vs. 63.7% ± 3.5, *p* = 0.006). Another group developed drug loaded finasteride microspheres (novel designed finasteride/poly (3-hydroxybutyrate-3-hydroxyvalerate)@polyvinyl alcohol/chitosan) tested in an animal model [76]. This approach demonstrated prolonged release of the drug for up to 51 days with an excellent biocompatibility profile. In vivo studies performed on rabbit ears did demonstrate evidence of ischemia due to the embolic effect. Another study evaluated embolization particles (PVA nanofibrous particles) loaded with finasteride compared to bland particles in a canine model [77]. Follow up imaging was performed at 1, 3- and 6-month intervals. At each time interval, significant differences were present between the groups. At the six months follow up, the crosslinked FNS/PVA nanofibrous particles had greater mean PV reduction compared to the bland crosslinked PVA nanofibrous particles group (83.9% vs. 69.2%, *p* < 0.05) suggesting the drug loaded particles did enhance the embolic effect.

## 8. Conclusions

Prostate artery embolization is a promising option for treating lower urinary tract symptoms in men with benign prostatic hypertrophy. Proper patient selection and thorough evaluation is critical to ensure clinical success. Many studies have shown clinical results that are comparable to the current surgical gold standard, TURP. The use of PAE offers similar results with fewer complications and fewer side effects, while eliminating the need for general anesthesia or hospitalization. Future studies comparing PAE to TURP and possibly other minimally invasive therapies will be critical in identifying exactly where PAE falls in the treatment algorithm for patients suffering from LUTS related to BPH.

## Figures and Tables

**Table 1:** Current surgical and minimally invasive treatment options for BPH. Prostate size range is as follows. small: <50 g, average: 50–80 g and large: >80 g [3].

Treatment	Description	Prostate Size
Open Simple Prostatectomy [4,5]	The complete or partial removal of the prostate via a suprapubic or retropubic approach.	Large
Transurethral Resection of the Prostate (TURP) [4,5]	A resectoscope is inserted via a transurethral approach. Portions of the excess prostate that are blocking the urine flow are cut and suctioned out.	Small to Average
Transurethral Incision of the Prostate (TUIP) [4,5]	A resectoscope is inserted via a transurethral approach. Small cuts are made at the area where the bladder and prostate are connected to increase urine flow.	Small
Transurethral Vaporization of the Prostate (TUVP) [4]	A small ball or button shaped instrument is used to heat the prostatic tissue, turning it into vapor and increasing urinary flow.	Small to Average
Transurethral Microwave Therapy (TUMT) [5]	A small antenna is inserted via a transurethral approach and delivers microwave thermal energy that destroys excess prostate tissue that is blocking urine flow.	Small to Average
Prostatic Urethral Lift (PUL) [4,6,7]	Small UroLift^®^ implants are placed in the prostate via a minimally invasive approach to lift and move the prostate, reducing the obstruction to urinary flow	Small to Large
Rezūm^™^ [5,7]	Uses water vapor thermal energy to ablate the prostate. The water vapor disrupts the prostatic cell membranes, resulting in necrosis of the prostatic cells. This reduces the size and volume of the prostate.	Small to Average
Aquablation [5,7,8]	Aquablation (AquaBeam^®^, Procept BioRobotics, Redwood Shores, CA, USA) uses a high-velocity water jet robotic instrument to remove prostatic tissue without the use of thermal energy. This is a minimally invasive, transurethral approach that utilizes ultrasound guidance.	Small to Average
Prostatic Stenting [9,10]	Temporary or permanent prostatic stents of various sizes can be placed in the urethra to resolve the urethral obstruction and improve the flow of urine.	Small to Large
Photoselective Vaporization of the Prostate (PVP) [4,5,7]	Uses laser energy via a transurethral approach to vaporize excess prostate tissue and improve the flow of urine by widening the urinary channel	Small to Average
Holmium Laser Enucleation of the Prostate (HoLEP) [4,5]	Uses a holmium laser via a minimally invasive, endoscopic treatment to enucleate prostatic tissue that is blocking urine flow. Next, an additional instrument is used to cut the excess prostatic tissue into smaller portions and remove them.	Small to Large
